# No Differences in 90-Day Complications and Admissions After Latarjet Procedure for Primary Bone Loss Versus Latarjet Procedure for Failed Arthroscopic Instability Repair

**DOI:** 10.1016/j.asmr.2022.06.010

**Published:** 2022-08-25

**Authors:** Neil Gambhir, Matthew G. Alben, Matthew T. Kim, Soterios Gyftopoulos, Andrew S. Rokito, Mandeep S. Virk

**Affiliations:** Division of Shoulder and Elbow Surgery, Department of Orthopaedic Surgery, NYU Langone Orthopedic Hospital–NYU Langone Health, New York, New York, U.S.A.

## Abstract

**Purpose:**

To investigate the variance in 90-day complication, emergency department (ED) visit, revision, and readmission rates between the Latarjet procedure (LP) performed as a primary procedure for the treatment of recurrent shoulder instability associated with critical levels of glenohumeral bone loss and the LP performed as a salvage surgical procedure after failed arthroscopic instability repair (FAIR).

**Methods:**

Patients who underwent a primary LP from 2016-2021 in a single surgeon’s practice were identified and divided into 2 cohorts based on the indication for surgery: primary LP for critical bone loss (unipolar or bipolar) (LP-PBL) or LP as salvage surgery for FAIR (LP-FAIR). Patients without a minimum follow-up period of 90 days were excluded. Chart review was conducted to analyze the prevalence of complications, ED visits and/or admissions, and secondary procedures in the 90-day postoperative period. Radiographic images were reviewed to evaluate for graft and/or hardware failure. An unpaired *t* test and the Fisher exact test were used to compare the 2 groups regarding continuous and categorical data, respectively, and the significance level was set at *P* < .05.

**Results:**

The final sample sizes consisted of 54 patients in the LP-PBL group and 23 patients in the LP-FAIR group. In the postoperative period, 4 complications were observed in the first 90 days. These included complex regional pain syndrome (n = 1) and superficial wound dehiscence (n = 1) in the LP-PBL cohort. Superficial suture abscess (n = 1) and audible crepitation (n = 1) were observed in the LP-FAIR cohort. There was 1 secondary intervention (arthroscopic debridement) in the LP-FAIR cohort. No statistically significant difference in complication rates, ED visits or admissions, or secondary procedures was found between the LP-PBL and LP-FAIR groups.

**Conclusions:**

The results of this study indicate that the 90-day complication, ED visit, revision, and readmission rates after open LP are low irrespective of the extent of glenoid or bipolar bone loss and history of arthroscopic instability repair.

**Level of Evidence:**

Level III, retrospective cohort study.

The Latarjet procedure (LP) has gained popularity in the past few decades as a treatment for anterior glenohumeral instability.^1^ Also known as a “coracoid transfer,” the LP is one of the most effective bone block operations used to treat recurrent shoulder instability (RSI) with critical bone loss (unipolar or bipolar).^2^^,^^3^ The LP involves the transfer of the coracoid process and conjoint tendon to the anteroinferior glenoid and provides stability via the triple-blocking effect, which includes a sling effect, restoration of the articular glenoid arc, and a capsular effect.^4^, ^5^, ^6^, ^7^

In the United States, arthroscopic Bankart repair (ABR) is the most common surgical procedure for the treatment of RSI.^1^ Although a technically well-performed ABR has a low instability recurrence rate, the presence of glenoid bone loss leads to markedly higher failure rates after ABR.^8^, ^9^, ^10^, ^11^ Although the LP is most commonly used for the treatment of RSI in the presence of critical bone loss, its indications have expanded as a primary intervention for high-risk shoulder instability patients (young male collision athletes) without critical bone loss and as a salvage operation for failed ABR or open instability repair.^7^^,^^12^, ^13^, ^14^

Recent studies have shown that LPs performed for failed arthroscopic instability repair (FAIR) have higher risks of redislocation and inferior clinical outcomes when compared with primary LPs performed for bone loss indications.^15^^,^^16^ Although studies have reported a 6% to 9% ninety-day complication rate in patients undergoing an open LP, our understanding of the difference in acute complication rates between its use as a primary intervention and its use as salvage therapy is limited.^17^, ^18^, ^19^ The purpose of this study was to investigate the variance in 90-day complication, emergency department (ED) visit, revision, and readmission rates between the LP performed as a primary procedure for the treatment of RSI associated with critical levels of glenohumeral bone loss and the LP performed as a salvage surgical procedure after FAIR.

## Methods

Institutional internal review board approval was granted for this retrospective study. Eighty patients who underwent an LP performed from 2016-2020 by the senior author (M.S.V.) were identified from an institutional database. Patients who underwent revision LPs or who did not have a minimum follow-up period of 90 days were excluded from this study. Patients were separated into 2 cohorts depending on whether the LP was performed to address instability arising from critical bone loss (LP-PBL) or the LP was performed as salvage surgery for FAIR (LP-FAIR).

### Data Collection

Manual chart review via Epic (Epic Systems, Verona, WI) was conducted by 3 clinical researchers (N.G, M.G.A, and M.T.K) to identify the complications, revisions, and readmissions that occurred within the first 90 days after surgery in both cohorts. Complications included but were not limited to hematoma, superficial or deep infection, wound complications (dehiscence), recurrent dislocation, thromboembolic complications (deep venous thrombosis or pulmonary embolism), neurologic complications including nerve palsy (axillary, musculocutaneous, or brachial plexus), and death. Remaining complications were classified as “other” for the purpose of analysis. Information on ED visits or admissions for any reason and secondary interventions in the first 90 days was also collected from electronic medical records. ED visits or admissions were further characterized as directly related versus unrelated to the procedure. In addition, radiographic images were reviewed to evaluate for graft and hardware failure. Any issues with data inclusion or exclusion were resolved after discussion with the senior author.

### Radiologic Analysis

Magnetic resonance imaging and computed tomography scans were available for analysis for all patients included in this study. The percentage of glenoid bone loss, Hill-Sachs lesion depth, and presence of off- or on-track lesions was determined for both groups. The glenoid bone loss percentage was estimated using the best-fit circle method on the provided sagittal images as previously described.^20^ Hill-Sachs lesion depth was calculated by reviewing the axial images and identifying the slice with the greatest degree of cortical impaction along the posterior humeral head. A best-fit circle was placed along the humeral head margins on this slice. Next, the depth was estimated by a measurement made between the impacted bone and adjacent circle margin. The presence of on- or off-track lesions was determined using previously described methodology.^21^

### Surgical Technique: Open LP

Patients in both cohorts underwent the LP in the beach-chair position under regional anesthesia (single-shot interscalene block) with few differences in the technique (described later). In brief, an 8-cm vertical incision is made from the coracoid toward the axillary fold. The cephalic vein is identified in the deltopectoral interval and is brought laterally with the deltoid. The clavipectoral fascia is incised, and the conjoint tendon is freed up distally until the level of the “three sisters.” The coracoacromial ligament is transected in its entirety, leaving a stump of tissue of approximately 10 mm on the coracoid. The attachment of the pectoralis minor tendon is then released subperiosteally from the medial coracoid, and a soft-tissue plane is developed between the conjoint tendon and the pectoralis minor distally.

A right-angled oscillating saw is used to perform the coracoid osteotomy at the junction of the horizontal and vertical parts of the coracoid. The undersurface of the osteotomized coracoid is then decorticated and flattened with a saw or burr. Two drill holes (3.5 mm typically) are placed in the graft using a freehand technique. A horizontal subscapularis split is performed at the junction of the upper two-thirds and the lower one-third of the subscapularis. A T-shaped capsulotomy is then performed, exposing the inferior half of the glenoid rim. The damaged anterior labrum and capsule are removed, and the anterior glenoid neck is decorticated with a burr or osteotome from the 3- to 6-o’clock position (right shoulder). In the LP-FAIR group, prior sutures and suture anchors were removed. Additionally, anterior glenoid rim fracture remnants were excised, if present, in both cohorts. The coracoid graft is then placed on the prepared surface of the glenoid and fixed with 2 screws. The stump of the coracoacromial ligament is repaired to the residual free capsular margin. A side-to-side repair of the subscapularis split is then performed, followed by a standard layered closure.

Postoperatively, a shoulder sling is used for the first 4 weeks, with pendulums and passive motion exercises beginning a few days after surgery. The subscapularis split, instead of a tenotomy, allows for the early introduction (2-4 weeks) of isometric strengthening of the rotator cuff and active range-of-motion exercises of the shoulder. Most patients are allowed to return to sport-specific activities by 4 to 6 months.

### Statistical Analysis

Statistical analysis was performed using SPSS Statistics (version 26.0; IBM, Armonk, NY). Descriptive statistics were calculated for both categorical and continuous variables. An unpaired *t* test was conducted to compare continuous data, and the Fisher exact test was conducted to compare categorical data. *P* < .05 was considered statistically significant. All calculated *P* values were reported as raw values, and the incidence of complications was reported as a percentage of the total sample of each cohort.

An a priori power analysis was conducted to determine sample size requirements for our study. We performed a power analysis based on an α value of .05 and an incidence of 6% for short-term complications of primary LP as previously reported.^17^ We conducted a power analysis for 2 independent proportions with the Fisher exact test, proportions of 6% versus 36% (6 times higher in the salvage group), statistical power of 80% (1 – β > 0.8), and a level of statistical significance of *P* < .05. This yielded a minimum sample size of 66 for the whole cohort.

## Results

A total of 77 patients (77 shoulders) were included in our study, of whom 54 were in the LP-PBL group and 23 were in the LP-FAIR group. Baseline patient demographic characteristics are shown in Table 1 and were remarkable for a statistically significant difference between cohorts with respect to body mass index (*P* = .049). No significant differences regarding the percentage of glenoid bone loss, Hill-Sachs lesion depth, or presence of off-track lesions were found between the groups (Table 2).

**Table 1:** Patient Demographic Characteristics

Characteristic	LP-PBL	LP-FAIR	*P* Value
Sample size, n	54	23	
Age, yr	30 ± 10	26 ± 6	.092
Male sex, n (%)	49 (90.7)	21 (91.3)	.094
BMI	27.1 ± 6.2	24.4 ± 2.79	.049∗
Current smoker, n (%)	11 (20.4)	4 (17.4)	.76

NOTE. Data are presented as mean ± standard deviation unless otherwise indicated.

BMI, body mass index; LP-FAIR, salvage Latarjet procedure after failed arthroscopic instability repair; LP-PBL, primary Latarjet procedure for critical glenohumeral bone loss.

∗ Statistically significant (*P* < .05).

**Table 2:** Radiologic Analysis

	LP-PBL	LP-FAIR	*P* Value
Sample size, n	54	23	
GBL, %	17 ± 7.9	15 ± 5.9	.65
HSL depth, mm	4.8 ± 2.8	4.6 ± 2.6	.80
Presence of off-track lesion, n (%)	26 (48)	9 (39)	.71

NOTE. Data are presented as mean ± standard deviation unless otherwise indicated.

GBL, glenoid bone loss; HSL, Hill-Sachs lesion; LP-FAIR, salvage Latarjet procedure after failed arthroscopic instability repair; LP-PBL, primary Latarjet procedure for critical glenohumeral bone loss.

A total of 4 postoperative complications occurred within the first 90 days after surgery. Two of these complications were seen in the LP-PBL cohort, which included 1 case of complex regional pain syndrome and 1 case of superficial wound dehiscence in the inferior 1 cm of the incision requiring local wound care and antibiotics. The patient with complex regional syndrome had a protracted recovery course with multispecialty treatment (pain management and occupational and hand therapy) for pain control and treatment of shoulder and hand stiffness.

In the LP-FAIR group, 2 patients experienced complications, which included a suture abscess and audible shoulder crepitus. The suture abscess was treated with local wound care and antibiotic treatment. The patient with audible crepitus from the joint was initially observed expectantly and underwent an extensive workup to rule out any mechanical impingement from hardware or bone graft. The patient eventually underwent arthroscopic debridement for symptomatic relief. Arthroscopic examination revealed that the graft was flush and healed. The screws were not prominent or close to the articular surfaces.

There were no 90-day ED visits or admissions for any cause in either cohort. There was 1 secondary intervention in the LP-FAIR group in the form of arthroscopic debridement and subacromial bursectomy for the patient with audible crepitus at approximately 3 months (102 days) after the index operation. When both cohorts were compared via an unpaired Student *t* test, no statistically significant difference was observed between groups for any category of complications, ED visits or admissions, or secondary procedures in the first 90 days after the open LP (Table 3).

**Table 3:** Complications and Secondary Interventions After Surgery

	LP-PBL (n = 54), n (%)	LP-FAIR (n = 23), n (%)	*P* Value
Complication			
Redislocation	0 (0.0)	0 (0.0)	—
Revision	0 (0.0)	0 (0.0)	—
Nerve palsy	0 (0.0)	0 (0.0)	—
Death	0 (0.0)	0 (0.0)	—
Infection	0 (0.0)	1 (4.3)	.299
Other	2 (3.7)	1 (4.3)	.528
ED visit	0 (0.0)	0 (0.0)	—
Readmission	0 (0.0)	0 (0.0)	—
Secondary intervention	0 (0.0)	1 (4.3)	.299

ED, emergency department; LP-FAIR, salvage Latarjet procedure after failed arthroscopic instability repair; LP-PBL, primary Latarjet procedure for critical glenohumeral bone loss.

## Discussion

In this study, we found no difference with respect to the incidence of 90-day complications between the LP-PBL cohort and LP-FAIR cohort. Furthermore, there were no differences between the 2 cohorts with respect to intraoperative complications or with respect to ED visits or readmissions in the first 90 days after the LP. There was 1 secondary intervention in the LP-FAIR group.

Although critical bone loss (unipolar or bipolar) is the most common indication for the LP in the United States, the LP has also been shown to be an effective salvage procedure after FAIR.^7^^,^^12^, ^13^, ^14^ Given that patients who have a history of failed ABR typically do not have the same extent of bone loss as instability patients with critical bone loss, there is a concern that these patients are at risk of a higher rate of postoperative complications in the setting of revision surgery. The presence of prior subscapularis tenotomy and/or prior hardware (metallic suture anchors), as well as the need for more extensive dissection, can predispose to complications such as subscapularis failure, hardware malposition, and hematoma. On the other hand, patients who have critical levels of bone loss typically have experienced higher numbers of dislocations and more chondral injury and are predisposed to a higher recurrence rate and residual pain.^15^^,^^16^ Despite these risks and predispositions associated with these 2 cohorts, we did not find any significant difference between the 2 groups with respect to 90-day complication, ED visit, readmission, or secondary surgical procedure rates.

The open LP has repeatedly shown excellent long-term results with low recurrence rates and high rates of return to sports.^2^^,^^3^ However, the complication rates associated with the LP have reportedly been found to be high and have ranged between 15% and 30%.^6^^,^^22^ Several recent studies have looked at complication and secondary intervention rates in the first 90 days after an open LP. A retrospective review of 441 patients by Scanlon et al.^23^ examined 90-day complications associated with the LP in a high-volume center. A complication rate of 4.3% was found, with hematoma formation (2%) being the most common complication. Hendy et al.^18^ conducted a similar retrospective review of 190 patients, reporting a complication rate of 9%, with graft failure (4.7%) being the most common complication, followed by nerve injury (3.2%). Intraoperative injury to the musculocutaneous, axillary, and suprascapular nerves has been reported as one of the major complications due to intraoperative events.^17^^,^^18^^,^^23^^,^^24^ Clinically, the long-term importance of this complication is mitigated as the literature describes reports of patients’ full recovery from these transient palsies.^24^ Screw-related complications were also a common occurrence in the work of Hendy et al., with divergent screws and single screws being predictors of early graft failure in the first 90 days after the open LP.

We believe that the aforementioned complications of the LP, to a large extent, are avoidable. In our series, hematoma formation, nerve palsy, graft fracture, and screw failure were not observed in the first 90 days after the LP in either cohort. We believe that some of the factors that account for the low complication rate are meticulous hemostasis, an adequate length of graft harvest (approximately 2 cm), and the use of screws that were appropriately sized and of superior quality. The complications encountered in our series were unusual (complex regional pain syndrome and audible crepitus) and not commonly reported.

### Limitations

Our study results should be interpreted with the following limitations: First, the study has a retrospective study design with unequal study groups. Second, this is a study of a single surgeon’s cases; this limits the generalized applicability of conclusions but at the same time provides a homogeneous sample without surgeon bias. Third, the study includes 90-day follow-up only and would miss important short-term follow-up differences in recurrence that may not be evident at 90-day follow-up.

## Conclusions

The results of this study indicate that the 90-day complication, ED visit, revision, and readmission rates after open LP are low irrespective of the extent of glenoid or bipolar bone loss and history of arthroscopic instability repair.

## Supplementary Data

ICMJE author disclosure forms
